# Thinking Outside of the Cereal Box: Breeding Underutilized (Pseudo)Cereals for Improved Human Nutrition

**DOI:** 10.3389/fgene.2019.01289

**Published:** 2019-12-20

**Authors:** Cody S. Bekkering, Li Tian

**Affiliations:** Department of Plant Sciences, University of California, Davis, Davis, CA, United States

**Keywords:** cereal, pseudocereal, nutrition, micronutrient, phytochemical, biofortification, phytonutrient

## Abstract

Cereal grains have historically played a critical role in sustaining the caloric needs of the human population. The major cereal crops, wheat, rice, and maize, are widely cultivated and have been subjected to biofortification to enhance the vitamin and mineral nutrient content of grains. In contrast, grains of several other cereals as well as non-grass pseudocereals are naturally rich in micronutrients, but have yet to be explored for broad-scale cultivation and consumption. This mini review focuses on the micronutrient and phytochemical profiles of a few emerging (pseudo)cereals and examines the current constraints of their integration into the global food system. Prospects of leveraging whole genome sequence information and modern breeding technologies to promote the breeding and accessibility of these crops are also discussed.

## Introduction

Among staple foods, cereal grains are advantaged for high starch content, relatively long-term storage capacity, and values as seed stocks. Wheat, rice, and maize constitute the major cereal crops that sustain over 50% of the caloric demand of the world population. Although these cereal grains make up a critical portion of many diets, they lack substantial amounts of micronutrients (vitamins and minerals) and phytonutrients (nutraceuticals and phytomedicines). Consequently, the hidden hunger due to micronutrient deficiency reportedly affects approximately 2 billion people globally (FAO, 2013), which raises the health concern regarding our heavy reliance on major cereal crops. To this end, multifaceted approaches including fortification, biofortification, and nutrient supplements have been deployed to ensure access to nutritious food, an important pillar of food security. On the other hand, some micronutrient and/or phytonutrient-rich (pseudo)cereal crops have historically taken on the role of a staple crop across many cultures, but are currently underutilized—having only percolated into small niches in the global food system. The present review examines the nutritional characteristics, cultivation, and germplasm collections of seven underutilized (pseudo)cereals. The limitations and opportunities for breeding and marketing these (pseudo)cereal grains for improving human nutrition are also discussed.

## Looking Beyond Staple Cereals and Into the Nutrient-Dense Underutilized (PSEUDO)Cereals

**Figure 1:**
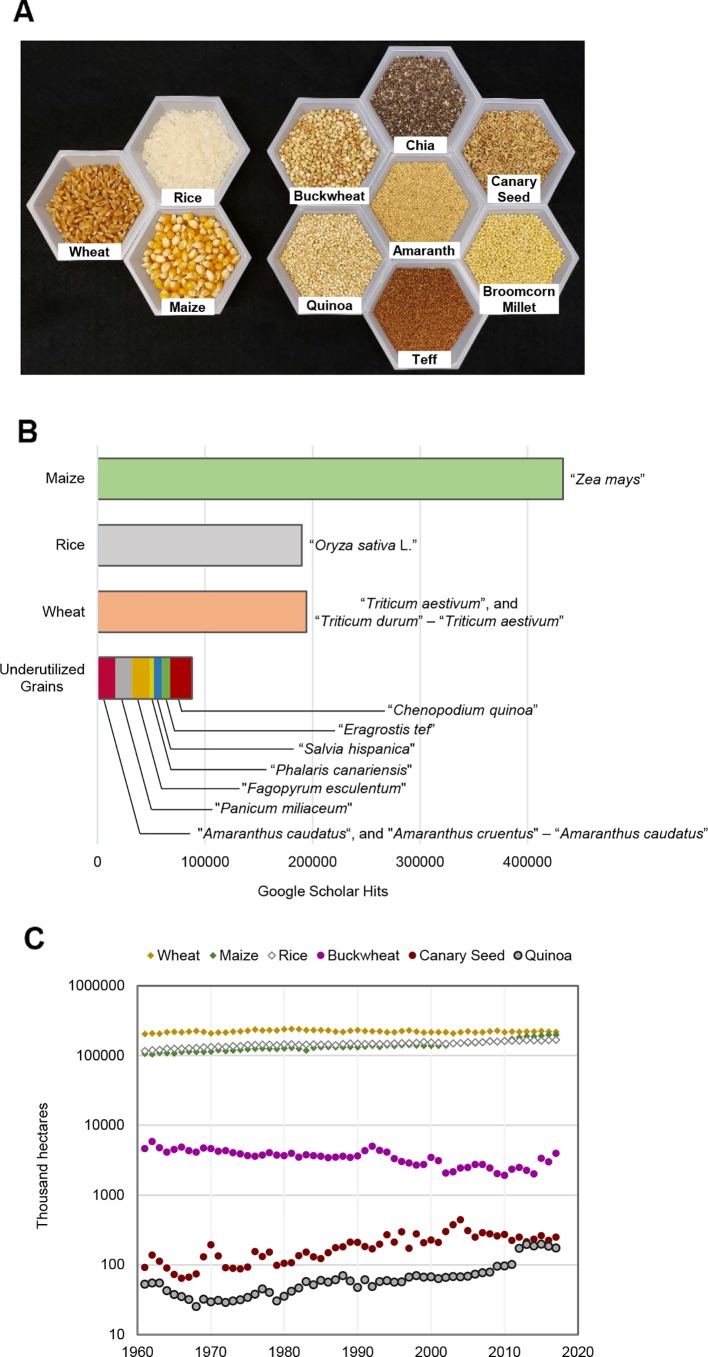
**(A)** Image of major staple cereal grains and seven selected underutilized (pseudo)cereal grains. **(B)** Google scholar hits from 2018 and before using the search terms indicated in the panel. When two search terms were used for a (pseudo)cereal grain, the not operator (-) was used in conjunction with the second search term to exclude results that also contain the first search term; the hits from the two searches were added. **(C)** Global growth acreages of major staple cereal grains and three selected underutilized (pseudo)cereal grains from 1961 to 2017. Data shown are Food and Agriculture Organization of the United Nations (FAO) aggregated estimates.

Like wheat, rice, and maize, broomcorn millet (*Panicum miliaceum* L.), canary seed (*Phalaris canariensis* L.), and teff [*Eragrostis tef* (Zuccagni) Trotter] are monocotyledonous plants in the family of Poaceae (grasses) (**Figure 1A**; **Table 1**). Amaranth (*Amaranthus* spp.), buckwheat (*Fagopyrum esculentum* Moench.), chia (*Salvia hispanica* L.), and quinoa (*Chenopodium quinoa* Willd), despite having seeds resembling the cereal grains, do not belong to Poaceae and are considered pseudocereals (**Figure 1A**; **Table 1**). Currently, these (pseudo)cereals or grain products are used as breakfast cereals, snacks, additions to salads, processed foods, flour, and beverages, etc., but not a substantial source of calories.

**Table 1:** Comparison of nutritional data and general characteristics of major staple cereal grains and seven selected underutilized (pseudo)cereal grains.

	Wheat	Maize	Rice	Broomcorn millet	Canary seed	Teff	Amaranth	Buckwheat	Chia	Quinoa
Nutritional data (per 100 g grain or grain flour)											
FDC ID	169761	170288	169756	169702	–	169747	170682	170687	170554	168874
Form Consumed	Wheat flour, white, all–purpose, unenriched	Whole grain, yellow	White, long–grain, unenriched	Whole grain	Whole groats	Whole grain	Whole grain	Whole–groat flour	Whole grain	Whole grain
Calories (kcal)	364	365	365	378	399	367	371	335	486	368
Carbohydrate (g)	76.31	74.26	79.95	72.85	68.7	73.13	65.25	70.59	42.12	64.16
Protein (g)	10.33	9.42	7.13	11.02	21.3	13.3	13.56	12.62	16.54	14.12
Total lipid (g)	0.98	4.74	0.66	4.22	6.7	2.38	7.02	3.1	30.74	6.07
Dietary Fiber (g)	2.7	7.3	1.3	8.5	6.2	8	6.7	10	34.4	7
Vitamin A, IU	0	214	0	0	–	9	2	0	54	14
Vitamin B–6 (mg)	0.044	0.622	0.164	0.384	–	0.482	0.591	0.582	–	0.487
Vitamin C (mg)	0	0	0	0	–	–	4.2	0	1.6	–
Vitamin E (mg)	0.06	0.49	0.11	0.05	–	0.08	1.19	0.32	0.5	2.44
Folate (µg)	26	19	8	85	100	–	82	54	49	184
Phosphorus (mg)	108	210	115	285	664	429	557	337	860	457
Potassium (mg)	107	287	115	195	400	427	508	577	407	563
Iron (mg)	1.17	2.71	0.8	3.01	6.6	7.63	7.61	4.06	7.72	4.57
Calcium (mg)	15	7	28	8	32	180	159	41	631	47
Zinc (mg)	0.7	2.21	1.09	1.68	3.7	3.63	2.87	3.12	4.58	3.1
Magnesium (mg)	22	127	25	114	216	184	248	251	335	197
**General characteristics**											
Group	Monocot	Monocot	Monocot	Monocot	Monocot	Monocot	Dicot	Dicot	Dicot	Dicot
Family	Poaceae	Poaceae	Poaceae	Poaceae	Poaceae	Poaceae	Amaranthaceae	Polygonaceae	Lamiaceae	Amaranthaceae
Center of origin	Middle East	Southern Mexico	Asia	Northern China	Mediterranean	East Africa	Mexico and Central America	Central Asia and Siberia	Guatemala and southern Mexico	Peru and Bolivia
Photosynthesis	C_3_	C_4_	C_3_	C_4_	C_3_	C_4_	C_4_	C_3_	–	C_3_
Sequenced genome	Yes (Appels et al., 2018)	Yes (Schnable et al., 2009)	Yes (Yu et al., 2002)	Yes (Zou et al., 2019)	No	Yes (Vanburen et al., 2019)	Yes (Clouse et al., 2016)	Yes (Zhang et al., 2017)	No	Yes (Jarvis et al., 2017)

Nutrition data in the uncooked, most commonly consumed form of the grains were obtained from the United States Department of Agriculture Food Data Central (https://fdc.nal.usda.gov/) with the exception of canary seed data, which were extracted from the nutritional factsheets published by Canaryseed Development Commission of Saskatchewan (https://www.canaryseed.ca/). Dashes indicate information not yet reported in the literature. FDC ID, Food Data Central identification number; IU, international unit.

Although these underutilized grains contain similar or lower starch contents than the staple cereal grains, they possess comparable or higher caloric values because decreases in carbohydrate content are offset by higher protein and lipid content (**Table 1**). The anatomy of the underutilized grains differs from the staple cereals in that they contain less endosperm (accumulating starch) and a higher proportion of embryos (accumulating proteins and lipids) (Prego et al., 1998; Valdivia-López and Tecante, 2015). It is noteworthy that higher caloric content, while a drawback in food systems of developed nations, is an asset in developing regions of the world where calorie deficiencies are a prevalent issue. Additionally, the higher protein content and more balanced amino acid composition of these underutilized grains is desirable. For instance, amaranth and quinoa grains are abundant in essential amino acids and showed a near optimal protein composition—one resembling that of cow milk (National Academy of Science, 1984). Bioactive peptides have also been found in amaranth and chia grains (Silva-Sánchez et al., 2008; Grancieri et al., 2019). Furthermore, the lack of gluten in these grains make them suitable for consumption by patients with coeliac disease.

With some exceptions, mineral nutrient content (potassium, phosphorus, magnesium, zinc, calcium, iron) of these underutilized grains is generally higher than that of their staple counterparts (**Table 1**). This discrepancy is as high as an order of magnitude in some cases (e.g., calcium in chia, amaranth, and teff). These underutilized grains are also more abundant in vitamins than white rice. In particular, grains of quinoa, canary seed, broomcorn millet, and amaranth are remarkably rich in folate (**Table 1**). Besides micronutrients, these underutilized grains also accumulate significant quantities of phenolic acids and flavonoids (phytonutrients) with antioxidant activities (Li et al., 2011; Gebremariam et al., 2014; Martínez-Cruz and Paredes-López, 2014; Zhang et al., 2014; Singh and Sharma, 2017; Tang and Tsao, 2017). However, some phytochemicals found in cereal grains (often located in husks), such as phytate, saponins, and tannins, are deemed as antinutrients because they tend to interfere with nutrient absorption and/or utilization.

## Current Limitations for Developing Underutilized (PSEUDO)Cereals

In spite of the potential advantages of more extensively leveraging these underutilized grain crops, several factors hinder the widespread incorporation of these crops into food systems and breeding regimes—factors that are bolstered by a relative lack of research into these crops (**Figure 1B**). These factors range from agronomical (growth acreage, yield potential), technological (trait improvement), social (knowledge diffusion), and economic (market buy-in), and have stark similarities regardless of the underutilized grain in question.

The agronomic potential of these underutilized grain crops is thus far poorly characterized. Grain crops grown outside of the plots of developed nations, such as quinoa, teff, chia, and amaranth, do not benefit from the high-input agriculture customary in the cultivation of major staple grains. As such, our knowledge of the yield and quality of these underutilized crops comes largely from low-input systems, limiting our ability to gauge their potential alongside major staple grains. Of the underutilized grains detailed here that benefit from high-input agriculture, such as broomcorn millet, buckwheat, and canary seed, their use is often constrained to that of a secondary crop—one grown to replace destroyed fields of staple crops or as a quick alternative to summer fallow. The short and less-than-optimal growth season allocated to these grains, while a sensible decision for a grower, hinders our ability to compare their yield and quality to their staple grain counterparts. This pattern of usage manifests in the low acreage of planting allocated to underutilized grains, magnitudes lower than major cereal crops (**Figure 1C**). Nevertheless, there was a gradual increase in quinoa production during the last few decades (**Figure 1C**).

Genetic limitations exist for some underutilized (pseudo)cereal crops. For instance, buckwheat is naturally cross-pollinated and exhibits self-incompatibility (Ueno et al., 2016). As such, it is necessary to develop self-compatible buckwheat lines for breeding and trait improvement. In addition, pipelines for mutagenesis and transformation are yet undeveloped and/or require optimization, resulting a reliance on natural variations for breeding in these grain crops. Currently, the intersection between genomics and breeding is also limited or nonexistent for these underutilized grains. Overall, underutilized grain crops are at present constrained by a lack of concerted breeding efforts committed to expanding their use in high-input agricultural systems.

Although cultivation and breeding knowledge exists in local communities for many of the underutilized grains noted here, the diffusion of this knowledge is often barred from reaching the broader global community of growers. In the case of chia, amaranth, teff, quinoa, and even buckwheat, both traditional knowledge and modern research trickles slowly across the language barrier into common languages used in global science and agriculture. This is especially pronounced for teff, where relevant information is commonly displayed only in Amharic. While language barriers do not exist in excess for broomcorn millet and canary seed, the diffusion of information about their cultivation is inhibited by their niche in the market. As broomcorn millet is used as birdseed outside of East Asia, and canary seed almost ubiquitously so, their cultivation has been restricted to growers with connections to distributers in the birdseed market—a market already possessing a limited demand.

Except for quinoa, the noted underutilized grains have thus far received little media and market attention. Without a considerable marketing effort, investment in a farm-to-fork pipeline for underutilized grains may prove unfruitful. Quinoa serves as an example of a marketing success in this regard. The endorsement of quinoa as a functional grain crop by high-visibility public figures contributed to its global spike in cultivation—a spike that was aided by the integration of growers into the global marketplace (**Figure 1C**) (Bazile et al., 2016). Other underutilized grains would need to overcome their marketing constraints to bring their cultivation and consumption out of obscurity and to establish a stronger foothold in the global market.

## Promises and Potential for Developing Underutilized (PSEUDO)Cereals

The above-mentioned limitations present a clear avenue for development that could bring with it many fruitful possibilities—an avenue with promise substantiated by ongoing scientific progress on these underutilized grains. Although the yields of underutilized grains are generally lower than staple grains, this could at least be partially attributable to the fact that these grains are often grown on less arable land with fewer inputs (e.g., teff, quinoa, amaranth, chia) or are briefly grown as cover crop to avoid summer fallow (e.g., buckwheat, broomcorn millet). Therefore, allotment of suitable cropland and growing seasons to underutilized grain crops can uncover their yield potential relative to the grains that serve as the cornerstone of global research, development, and consumption.

The classic breeding methods remain applicable and valuable to these underutilized grains. Except for the limited germplasm collections for chia (Bochicchio et al., 2015) and canary seed (Cogliatti et al., 2011), there are over 3,000 accessions reported for quinoa (FAO, 2011; FAO, 2013), 5,000 accession for teff (Assefa et al., 2015), more than 10,000 accessions for buckwheat (Zhou et al., 2018), over 29,000 accessions for broomcorn millet (Vetriventhan et al., 2019), and at least 61 collection centers for amaranth (Das, 2016). Comprehensive evaluation and characterization of these germplasm collections will provide critical resources for breeding high-yield, elite crop varieties. To this end, next-generation sequencing technologies can be utilized to examine the genetic diversity of germplasms that have been adapted to different regions and production environments. Additionally, whole-genome sequencing (WGS) data of the germplasm collections encompass a broad range of genomic variants and can boost the power of genomic prediction (Hickey et al., 2017). Besides natural variations, the genetic diversity of the breeding population for the underutilized grains can be further enhanced through physical and chemical mutagenesis.

The emergence of genomic information for buckwheat, broomcorn millet, quinoa, amaranth, and teff pave the way for the development of breeding pipelines for desirable traits in the post-genomic era—pipelines that can integrate the emerging omics, phenotyping, and genome editing technologies (**Table 1**). These available reference genomes facilitate not only WGS in genotyping, but also discovery of genes, single nucleotide polymorphisms (SNPs), and genomic structural variants. High throughput genotyping coupled with high throughput phenotyping (phenomics) and crop modeling will enable acquisition of valuable trait data to assist in breeding. The genome sequences also allow precise and effective genome editing of target genes (Chen et al., 2019). With the exception of canary seed, there have been reports on genetic transformation and regeneration of these underutilized (pseudo)cereal plants (Jofre-Garfias et al., 1997; Eisa et al., 2005; Plaza-Wüthrich and Tadele, 2012; Gebre et al., 2013; Marconi et al., 2013; Suvorova, 2016). Although the efficiency of plant transformation remains to be optimized, it enables delivery of the genome-editing system to these crops. The underutilized (pseudo)cereals are reportedly tolerant/resistant to biotic and abiotic stresses that threaten crop production, such as insects, pathogens, weeds, drought, high temperature, UV-B radiation, heavy metal contamination, as well as salinity, alkalinity, acidic, or low fertility in soil (Assefa et al., 2015; Habiyaremye et al., 2017; Hinojosa et al., 2018). Genomic analyses have already begun to associate stress tolerance/resistance to molecular and physiological responses. Understanding the underlying mechanisms of stress tolerance in these underutilized cereals will also be useful for breeding other agronomically and economically important crops.

There is promise for these underutilized (pseudo)cereals in the marketing sector as well. The success of quinoa in being marketed as a functional grain crop with a rich history has laid the groundwork for other grain marketers to follow suit. Even outside the grains, functional foods are increasingly sought after in global markets, with clear parallels being visible in the western markets of avocado, kale, pomegranate, and wine. Globally, marketing these grain crops as a nutritious source of carbohydrates could promote their import to developing regions—a treatment that even quinoa could benefit from. Marketing and subsequent supply chain reconfiguration should of course proceed such that local demand for the traditional crops is still satisfied, as a leading criticism of the rapid adoption of quinoa was the resulting lapse in quinoa consumption by the locals that had depended on it for generations (Friedman-Rudovsky, 2012).

Promises in marketing are substantiated by the fact that most of these grains are sold and consumed either as whole grains or whole grain products (**Table 1**), a form that retains the nutrient content (without losing it to postharvest processing) and fits easily into the functional food space of western markets. The exceptions to this are buckwheat, canary seed, and broomcorn millet. Buckwheat is almost exclusively sold as dehulled grouts and flour produced from dehulled groats. Canary seed’s potential in the human diet has been elucidated thus far for dehulled groats also (Abdel-Aal et al., 2011; Mason et al., 2018), while broomcorn millet is occasionally sold as white flour. The additional processing steps reduced the mineral nutrient content in dehusked buckwheat grains as observed similarly in the major cereals (Oghbaei and Prakash, 2013; Pandey et al., 2015). On the other hand, these additional steps in processing could have unstudied roles in removing antinutritional phytochemicals from these grains as well, much like the saponin removal steps in quinoa production (Jarvis et al., 2017). Thorough examination of the role that postharvest processing could have for antinutrient mitigation in other underutilized grain crops could aid in their wider application while simultaneously providing yet another selling point to leverage in marketing.

## Perspectives

Although it is not envisioned that the underutilized grains will play a major role as food staple in the near future, an expansion of their cultivation and utilization will build nutritional synergy with the major cereal grains. Climates non-conducive to staple crop cultivation such as hot semi-arid, subtropical highland, and arid subtropical could be leveraged for food production, contingent on investment in the biology and marketing of these underutilized crops. A diversity of photosynthetic modes in the underutilized grains substantiates this potential for broader cultivation—with the existence of C_4_ species removing the need for extensive engineering efforts such as those carried out in rice (**Table 1**).

By leveraging available germplasm collections and expanding genetic resources, climate-adapted elite varieties can be developed for the underutilized grain crops. Increased understanding of the genetic underpinnings of many plant traits such as lodging resistance, seed size, grain shattering, and stress tolerance/resistance, as well as development of advanced techniques such as mechanical harvesting, food processing, and postharvest storage, will bring into focus clear avenues for improvement of underutilized grains that can be pursued through strategic plant breeding. In addition, making innovative breeding technologies and integrated plant breeding platforms accessible to local breeders and small farmers is essential for implementation of these breeding strategies. Furthermore, international collaborations and partnerships, such as the African Orphan Crops Consortium (AOCC) (Hendre et al., 2019), will accelerate the development of the climate-resilient (pseudo)cereals. Overall, complementary to biofortification of major cereal grains, better utilization of underutilized grains in diet will have far-reaching impact on alleviating the burden of the hidden hunger crisis.

## Author Contributions

CB and LT conceived and wrote the review.

## Funding

We thank USDA-NIFA (grant 2017-67013-26164 to LT) for supporting our work on provitamin A biofortification of wheat grains.

## Conflict of Interest

The authors declare that the research was conducted in the absence of any commercial or financial relationships that could be construed as a potential conflict of interest.
